# Be Friendly, Stay Well: The Effects of Job Resources on Well-Being in a Discriminatory Work Environment

**DOI:** 10.3389/fpsyg.2018.00413

**Published:** 2018-04-03

**Authors:** Donatella Di Marco, Alicia Arenas, Gabriele Giorgi, Giulio Arcangeli, Nicola Mucci

**Affiliations:** ^1^Business Research Unit, University Institute of Lisbon, Lisbon, Portugal; ^2^Department of Social Psychology, Faculty of Psychology, University of Seville, Seville, Spain; ^3^Department of Human Sciences, European University of Rome, Rome, Italy; ^4^Department of Experimental and Clinical Medicine, University of Florence, Florence, Italy

**Keywords:** discriminatory work environment, workers’ well-being, job resources, job autonomy, social support, occupational medicine

## Abstract

Many studies have focused on the negative effects of discrimination on workers’ well-being. However, discrimination does not affect just victims but also those people who witness discriminatory acts or who perceived they are working in a discriminatory work environment. Although perceiving a discriminatory work environment might be a stressor, the presence of job resources might counteract its negative effects, as suggested by the Job Demand-Resources model. The goal of this study is to test the effect of perceiving a discriminatory work environment on workers’ psychological well-being when job autonomy and co-workers and supervisor support act as mediator and moderators respectively. To test the moderated mediation model data were gathered with a sample of Italian 114 truckers. Results demonstrated that job autonomy partially mediates the relationship between perceiving a discriminatory work environment and workers’ well-being. Main interactional effects have been observed when co-workers support is introduced in the model as moderator, while no main interactional effects exist when supervisor support is introduced. Theoretical and practical implications are discussed.

## Introduction

It is well-known that current societies and workplaces are becoming more diverse, due to several reasons, such as the shortening of geographical distances and the free movement of people (Arenas et al., 2017). Even in those countries that have developed a legislative framework which protects people who belong to vulnerable groups, discrimination is still a contemporary issue to solve. According to the last report on Discrimination in European Union (EU) (European Commission, 2015), European people think that discrimination on the grounds of ethnic origins is widespread in EU (64%), followed by sexual orientation (58%), gender identity (56%), religion beliefs (50%), and disabilities (50%). And the list might continue. Data about equal opportunities in employment are not better, being elder people (56%), people who belong to an ethnic minority (46%), and people with disabilities (46%) those are perceived as the most discriminated against during the recruitment process (European Commission, 2015).

The negative effect of discrimination is not suffered only by victims. Past research has shown that witnessing discriminatory behaviors or perceiving a discriminatory work environment might have negative effects on workers’ well-being (Trau, 2015; Di Marco et al., 2016). In fact, workers might perceive the work context as hostile and be afraid about being the next victim.

Although perceiving a discriminatory work environment might constitute a stressor, organizations can provide instrumental or affective resources (e.g., job autonomy, social support, etc.) able to alter its adverse effects.

The goal of this article is trying to discover the role of job autonomy (as mediator) and social support (as moderator) in the relationship between perceiving a discriminatory work environment and workers’ well-being. In order to validate the role of such job resources a moderated-mediation model will be tested. Such relationships will be analyzed with a specific group of workers: truck drivers. Although much research has focused on people employed in this occupation, most of it analyzed truck drivers’ health in terms of sleeping problems (Braeckman et al., 2011), health knowledge (Versteeg et al., 2018), fatigue (Meng et al., 2016), food habits (Hamilton and Hagger, 2018), etc. However, to our knowledge, there are not studies that analyzed the effect of perceiving a discriminatory work environment on well-being. In fact, although most of the time truck drivers do not share a physical work environment with other co-workers, they maintain contact with supervisors or colleagues at the head office; moreover, they are constantly in contact with customers (Occupational Information Network, 2018).

This article offers three main contributions: firstly, it allows advancing in the field of discriminatory behaviors at work, explaining some mechanisms and factors that might alter the negative effects of discrimination on workers’ well-being. Secondly, it sheds light on the effectiveness of the resources based interventions, showing that not all types of resources are useful to counteract specific demands. Finally, it offers to human resource managers tools to counteract the prejudicial effects of discrimination at work.

### Affected by a Discriminatory Work Environment: The Prejudicial Effects on Well-Being

The negative effects of discrimination on victims’ well-being have been explored by several studies (Pascoe and Smart Richman, 2009; Volpone and Avery, 2013; Ek et al., 2014; Di Marco et al., 2015; Welbourne et al., 2015; Halim et al., 2017; Medina and Gamero, 2017). Discrimination is a stressor which reduces physical and psychological health, produce chronic pain, reduce self-esteem, decreases job satisfaction, and increases job tension (Ensher et al., 2001; Paradies, 2006; McGonagle and Hamblin, 2014).

Research on discrimination distinguishes between overt and covert (subtle) forms of discrimination (Hebl et al., 2002; Chao and Willaby, 2007; Jones et al., 2016). Given the widespread undesirability of discriminatory behaviors, specially in those countries where vulnerable groups are protected at a legislative level, people try to behave in an egalitarian way (Cortina, 2008), particularly when internally motivated by beliefs about equality (Butz and Plant, 2009). However, even those people who believe in equality, might still hold prejudice and negative stereotypes at an unconscious level. Thus, negative attitudes against some groups might be expressed by means of subtle discriminatory acts (Dovidio, 2001; Cortina, 2008). Recently, a multidimensional framework for explaining discrimination recognizes three different continuums that entail subtlety, formality, and intentionality (Jones et al., 2017). Discriminatory behaviors might be subtle vs. overt; formal (work-related) vs. informal (interpersonal); and with a clear vs. ambiguous intention to harm the victim.

Discrimination is a stressor for victims, but also for bystanders. Past research has shown that witnessing discrimination or perceiving a discriminatory work environment might be prejudicial for workers’ well-being (Schmader et al., 2012; Di Marco et al., 2016). Even those people who do not belong to any protected groups might feel threatened by such behaviors, perceiving an unsafe work environment. Therefore, according to previous research, perceiving a discriminatory work environment is also considered a stressor (Di Marco et al., 2016), a demand which affects negatively workers’ well-being.

However, the negative effects of perceiving a discriminatory work environment might be counteracted by organizations by offering other resources able to eliminate or mitigate the effects of such stressor. Such process might be understood through the lens of the Job Demand-Resources (JD-R) Model (Demerouti et al., 2001), a theoretical framework which categorizes organizational factors in two wide groups: demands and resources. Demands are those factors which entail an emotional or cognitive effort and produce consequences at a physical or psychological level. For instance, workload, work pressure, and role ambiguity are considered demanding factors. As stated before, perceiving a discriminatory work environment is also a demand. However, according to the JD-R Model, the negative effects of high demands might be counteracted by the presence of resources, which constitutes a set of emotional or physical factors that help workers to achieve their goal, reduce the costs associated to organizational demands, and motivate people to personal development (Bakker and Demerouti, 2006). Might job resources be useful when workers perceiving a discriminatory work environment?

### The Role of Job Resources in a Discriminatory Work Environment

Job resources are useful to counteract the negative effects of high job demands (Bal et al., 2017), but they are also important *per se*. For instance, co-workers’ and supervisors’ support, or instrumental resources are considered job resources (Demerouti et al., 2001). Therefore, job demands and job resources interact. Although job demands and job resources covary, in a recent work Bakker and Demerouti (2017) remember that the sign of the correlation between them is a research question for future studies. It depends on several factors, such as the work sector, the hierarchical position, etc. For instance, people in a managerial position might face more demands but they would also have more resources available to cope with them (Bakker and Demerouti, 2017). Moreover, it might depend on the type of demands that workers have to face. In the following sections we are going to examine two types of resources that might counteract the negative effects of perceiving a discriminatory work environment.

#### The Mediating Role of Job Autonomy

Job autonomy, in terms of workers’ degree of decisional power about how and when develop their tasks (Parker, 2014), is considered as a job resource. The concept of job autonomy entails several dimensions, given that it is possible to have autonomy in terms of when, how and with which means developing a task (Morgeson and Humphrey, 2006). Several studies have demonstrated the positive outcomes of job autonomy: it improves well-being, job satisfaction, and motivation; it also diminishes job exhaustion, turnover intentions and work-family conflict (Spector, 1986; Clark, 2002; Kossek et al., 2006; Fernet et al., 2012; Park and Searcy, 2012; Gaille, 2013; Kubicek et al., 2017).

However, perceiving a discriminatory work environment might inhibit people from using job autonomy or might have detrimental effect on the perception of job autonomy. For instance, past studies on sexism showed that women who perceive workplace sexism also perceive less job autonomy (Manuel et al., 2017). Therefore, if discrimination might reduce the perception of job autonomy, it also might decrease the positive effects of such job resource.

Moreover, past studies have considered the mediating role of job autonomy. Previous research (Fernet et al., 2012) has demonstrated that job autonomy mediates the relationships between work overload and emotional exhaustion, between social support and emotional exhaustion, and between job control and emotional exhaustion. When workers perceive a high level of job autonomy, they might feel free to avoid those situations that are perceived as more discriminatory, given the flexibility they have in terms of how, when, and where develop their task. Therefore, a high perception of job autonomy might contribute to eliminate the negative effects of perceiving a discriminatory work environment on workers’ well-being:

Hypothesis 1: Workers’ perception of job autonomy will mediate the relationship between perceiving a discriminatory work environment and workers’ well-being.

#### The Buffering Effect of Social Support

Another job resource traditionally studied is social support, a multidimensional construct that entails several facets (Bowling et al., 2004). It refers to psychological and material resources that people receive from their network in order to overcome stressful situations (Cohen, 2004). People might perceive emotional support, instrumental, and informational support. At the workplace, such types of support are conveyed by the feeling that colleagues and supervisors care for one (emotional support); that the others are open to help one in development of his/her tasks (instrumental support); and that the others can provide important information to cope with stressful situations (informational support) (Viswesvaran et al., 1999; Cohen, 2004).

As well as job autonomy, social support is considered a job resource (Bakker and Demerouti, 2006). Several studies have analyzed the effect of social support on workers’ well-being (Cohen, 2004; Kossek et al., 2011). In line with such studies, perceiving social support is important in reducing the negative effects of stressful situations, by diminishing the level of threat perceived by a person. Perceiving support diminishes job strain, moderates the effect of stress on psychological well-being, and increases job satisfaction (Kawachi and Berkman, 2001; Cohen, 2004; Caesens et al., 2014; Blanch, 2016).

Social support, at organizations but also in the daily life, is a resource that might counteract the prejudicial effects of being victim of discrimination (Brondolo et al., 2009; Ajrouch et al., 2010; O’Brien et al., 2016). A study developed with African–American mothers demonstrated that perceiving everyday discrimination produce psychological distress that might be buffered by emotional support (Ajrouch et al., 2010). Social support has also been studied as a coping strategy applied by those people who are victim of discrimination (Brondolo et al., 2009).

Given the buffering effect of social support on workers’ well-being when they are victims of discrimination, we hypothesize that social support will also reduce the negative effects of perceiving a discriminatory work environment on well-being.

Two specific sources of social support at the workplace are co-workers and supervisors, support. Several studies have shown the buffering effect of supervisor support on the relationship between being victim of discrimination and well-being (e.g., O’Brien et al., 2016) but less it is known about the buffering role of co-workers support. However, the role of bystanders has been recognized as important in other types of mistreatment, such as bullying: witnesses might offer a valid support, although they are not always able to intervene (D’Cruz and Noronha, 2011; Mulder et al., 2014).

For this reason, we are going to test separately the buffering effect of supervisors and co-workers support:

Hypothesis 2a: Co-workers’ support will moderate the relationship between perceiving a discriminatory work environment and workers’ well-being: the more people receive co-workers support, the less perceiving a discriminatory work environment will affect workers’ well-being.

Hypothesis 2b: Supervisor support will moderate the relationship between perceiving a discriminatory work environment and workers’ well-being: the more people receive supervisor support, the less perceiving a discriminatory work environment will affect workers’ well-being.

## Materials and Methods

### Participants and Procedure

Participants were 114 Italian truckers. The survey was carefully administered by a psychologist, ensuring anonymity, and privacy rules as well as helping the full comprehensions of items. The time required to administer the test to each individual was 30 min. Administration was carried out by common agreement at the end of the working day in groups that did not exceed 4/5 units.

It is not surprising that all participants were men, given that truck industry is a sector prevalently male dominated (Lichtenstein et al., 2008). Only a minor group (3.5%) holds a permanent contract, while the majority (95.5%) had temporary contract. 65.8% of participants have worked at the organization less than 15 years, while 34.2% have worked for a longer period.

Data were collected through a self-reported questionnaire and participants were informed about the anonymity and confidentiality of the survey.

### Measures

#### Discriminatory Environment (α = 0.79)

Participants answered the subscale of the Stress Questionnaire (SQ) developed by Giorgi et al. (2013). The subscale comprises seven items which measure to what extent people think that their organization discriminate on the bases of race, age, sexual orientation, religion, disabilities, or ideology. Responses were scored on a five-point Likert scale ranging from 1 (strongly disagree) to 5 (strongly agree). An example of item is “People in this organization may be exposed to stress or risks to a greater extent because of their sexual orientation.”

#### Psychological Well-Being (α = 0.70)

The 12-item Goldberg’s (1972) General Health Questionnaire, developed by Fraccaroli et al. (1991), was used. The scale comprises 12 items which measure perceptions about participants’ psychological well-being in the last weeks. Items were rated according to a four-point scale, from 0 (less than usual) to 3 (much more than usual). Higher score evidences a higher degree of psychological distress; therefore, participants’ final results in this scale may oscillate between a minimum of 0 points and a maximum of 36 points. An example of an item is “You feel unhappy and depressed.”

#### Supervisor Support (α = 0.76)

A subscale of the SQ developed by Giorgi et al. (2013) was used. The subscale comprises four items which measure to what extent workers receive help and support from their supervisors. Responses were scored on a five-point Likert scale ranging from 1 (strongly disagree) to 5 (strongly agree). An example of item is “I can count on my supervisor when I have a problem at work.”

#### Co-workers Support (α = 0.67)

Participants answered a subscale of the SQ developed by Giorgi et al. (2013). The original subscale comprises five items which measure the level of co-workers support perceived by participants. Since the reliability is higher if the item “It is difficult to receive the help of my colleagues in a difficult moment” is eliminated, we only use four items. Responses were scored on a five-point Likert scale ranging from 1 (strongly disagree) to 5 (strongly agree). An example of item is “I receive the help and support I need from my co-workers.”

#### Job Autonomy (α = 0.70)

A subscale of the SQ developed by Giorgi et al. (2013) was used. The original subscale comprises five items which measure to what extent workers perceive autonomy when they carry out their work. Since the reliability is higher if the item “I can decide when having a break” is eliminated, we only use four items. Responses were scored on a five-point Likert scale ranging from 1 (strongly disagree) to 5 (strongly agree). An example of item is “I can plan and program my work.”

#### Sociodemographic Data

Participants also reported sex (female or male), seniority (less than 15 years or more than 15 years), and type of contract (permanent or temporary).

### Analyses

Pearson correlation analyses were carried out to explore the association between variables included in the study. In order to test the mediational power of the job autonomy in the relationship between perceiving a discriminatory work environment and health (H1), we use PROCESS macro for SPSS (model 4) (Hayes, 2013). To test the buffering effect of co-workers support and supervisor support on the third path (perceiving a discriminatory work environment – psychological well-being) of the mediation model above described, we performed the model 5 of the PROCESS macro for SPSS.

## Results

**Table 1** presents means, standard deviations and correlations of all the variables considered in our model.

**Table 1:** Means, standard deviations, and intercorrelations among variables.

	*M*	*SD*	1	2	3	4	5
(1) Discriminatory work environment	2.18	0.79	-				
(2) Psychological well-being	19.66	3.70	0.42^∗∗^	-			
(3) Supervisor support	4.01	0.85	-0.39^∗∗^	-0.23^∗^	-		
(4) Co-workers support	3.60	0.73	-0.30^∗∗^	-0.37^∗∗^	0.15	-	
(5) Job autonomy	3.84	0.64	-0.24^∗∗^	-0.36^∗∗^	0.21^∗^	0.38^∗∗^	-

*N = 114; ^∗^*p* < 0.05; ^∗∗^*p* < 0.01.*

As stated before, higher rates of psychological well-being mean psychological distress. For this reason, the relationship between experiencing a discriminatory work environment and psychological well-being is positive, and also significant. Moreover, perceiving a discriminatory work environment is significantly related with the job resources involved in this study: job autonomy, co-workers support, and supervisor support. In these cases, the relationship is negative. Truckers’ psychological well-being correlates negatively with all the job resources present in the study. Thus higher rates of psychological distress are associated with lower rates of job autonomy, co-workers support, and supervisors support.

To test our hypotheses, the model 4 of PROCESS macro for SPSS (Hayes, 2013) was used. Firstly, the mediation hypothesis was tested (H1) and it resulted partially confirmed. In fact, results show that perceiving a discriminatory work environment is negatively and significantly related with job autonomy (*p* < 0.01; *R*^2^ = 0.06). Job autonomy is also significantly and negatively related with psychological well-being (*p* < 0.01; *R*^2^ = 0.25). Therefore, people with higher rates of job autonomy will report less psychological distress. On the contrary, perceiving a discriminatory work environment is related positively and significantly with workers’ psychological well-being, enhancing their psychological distress (*p* < 0.01; *R*^2^ = 0.18). However, when job autonomy is introduced as mediator the effect of perceiving a discriminatory work environment on psychological well-being is reduced (*p* < 0.01; *R*^2^ = 0.06) but still significant (see **Table 2**). For this reason, H1 is partially confirmed.

**Table 2:** Regression results for mediation.

Variable	*b*	*SE*	*t*	*P*	LLCI	ULCI
**Direct and total effects**						
JA regressed on D (a)	-0.19	0.07	-2.62	0.009	-0.34	-0.05
PWB regressed on JA, controlling D (b)	-1.58	0.49	-3.22	0.001	-2.55	-0.61
PWB regressed on D, controlling JA (c)	1.97	0.40	4.93	0.000	1.18	2.77
PWB regressed on D (c′)	1.66	0.40	4.21	0.001	0.88	2.45

	**Value**	***SE***	***z***	***P***		

**Indirect effect and significance using normal distribution**						
Sobel	0.31	0.15	1.98	0.047		

	***M***	***SE***	**LLCI**	**ULCI**		

**Bootstrap results for indirect effect**						
Effect	0.31	0.21	0.02	0.89		

*N = 114. D, discriminatory work environment; JA, job autonomy; PWB, psychological well-being; LL, lower limit; UL, upper limit; CI, confidence interval. Unstandardized regression coefficients are reported. Bootstrap sample size = 10,000.*

The resampling procedure (10,000 bootstrap samples) indicates a significant indirect effect, since the confidence interval at 95% does not include the value zero (*k*^2^ = 0.07; bootstrapped 95% CIs of 0.01–0.17) (Preacher and Hayes, 2008). Our mediation model explains 25% of employees’ psychological well-being variance [*F*(2,111) = 18.37; *p* < 0.01].

To test the buffering effect of the co-workers (H2a) and supervisor support (H2b) on the relationship between perceiving a discriminatory work environment and psychological well-being, mediated by job autonomy, we performed the model 5 of PROCESS macro for SPSS (Hayes, 2013). Results partially supported H2a. However, although experiencing a discriminatory work environment has an indirect effect on workers’ psychological well-being through job autonomy, bootstrap CIs showed that this effect does not exist with higher level of co-workers support (see **Table 3**).

**Table 3:** Moderated mediation analysis for discriminatory work environment, job autonomy, psychological well-being, and co-workers support.

Predictor	*b*	*SE*	*t*	*p*	LLCI	ULCI
**Job autonomy**						
Constant	3.83	0.06	64.74	0.000	3.72	3.95
D	-0.19	0.09	-2.05	0.427	-0.38	-0.01
**Psychological well-being**						
Constant	23.33	1.95	11.97	0.000	19.47	27.19
JA	-1.03	0.48	-2.13	0.035	-1.20	-0.07
D	1.17	0.34	3.38	0.001	0.48	1.85
CWS	-0.77	0.38	-2.05	0.042	-1.52	-0.03
D × CWS	-1.78	0.74	-2.41	0.017	-3.25	-0.32

**Values^a^**	**Conditional indirect effect at values of JA**					
**Co-worker support**	**Boot indirect effect**	**Boot SE**	***p***	**LLCI**	**ULCI**	

-0.73	2.47	0.69	0.000	1.10	3.84	
0.00	1.17	0.34	0.001	0.48	1.85	
0.73	-0.13	0.59	0.823	-1.29	1.03	

*N = 114. ^*a*^Range of values of the output provided by the macro. D, discriminatory work environment; CWS, co-workers support; JA, job autonomy; PWB, psychological well-being; LL, lower limit; UL, upper limit; CI, confidence interval. Unstandardized regression coefficients are reported. Bootstrap sample size = 10,000.*

Results do not support H2b since main interaction effects were not found (*B* = -0.49, *SE* = 0.50, *p* = 0.335). Therefore, supervisor support does not buffer the relationship between perceiving a discriminatory work environment and psychological well-being.

## Discussion

This study aimed to understand the role of job resources when a discriminatory work environment is perceived by workers. In line with previous studies, we confirm that job autonomy is an important resource connected with psychological well-being (Park and Searcy, 2012). Results also showed that job autonomy partially mediates the relationships between perceiving a discriminatory work environment and workers’ psychological well-being. Therefore, people who can decide how, when and where developing their tasks might feel less affected at psychological level by a discriminatory work environment, since they can avoid it.

Moreover, this study explores the buffering effect of social support (from co-workers and supervisor) in the relationship between discriminatory work environment and psychological well-being, mediated by job autonomy. Our results showed that a main interaction exists when co-workers support is taken into account, but it does not in the case of supervisor support. Thus, co-workers help in coping with the negative effects of a discriminatory work environment when workers also experiment job autonomy.

Although a negative correlation exist between supervisor support and perceiving a discriminatory work environment, this specific job resource is not helpful when workers perceive a discriminatory work environment. This result was unexpected, given the role of supervisor support in buffering the relationship between discrimination and well-being (O’Brien et al., 2016). However, we have to consider that our sample is composed by truckers, people who might spend the majority of their time traveling and who might maintain few contacts with their supervisors. Therefore, in this specific job, co-workers support might be more powerful in coping with a discriminatory work environment than supervisors support. Moreover, truck drivers might have more autonomy than workers in other occupations. According to that, it is also possible that people who share a physical work environment with other colleagues might be more affected by the perception of a discriminatory work environment. Thus, future studies should reply this moderated mediation model with workers of other sectors.

We have also seen that higher levels of co-workers support do not work as moderator. Thus higher levels of co-workers support, in a model where job autonomy is also present, might have a non-linear effect on workers psychological well-being. Future studies should explore this possibility and longitudinal studies should be carry out (Boyd et al., 2011).

Resources based interventions are useful tools to improve people’s work experience. However, as other researchers have highlighted (Baumeister and Alghamdi, 2015), this kind of interventions need to be adjusted to a specific target. Therefore, although we have seen that supervisor support does not moderate the mediation model we tested with truckers, it represents a powerful resource that might contribute to the well-being of workers of others sectors/job positions. Moreover, the effects of resources need to be understood within the JD-R Model. As stated above, job demands and resources covary (Bakker and Demerouti, 2017), but the sign of their relationship depends by many factors (e.g., hierarchical position, sector, etc.). Therefore, higher levels of job resources do not mean working under lower demands and *vice versa*. This might explain why higher levels of co-workers support are not useful to buffer the relationship between perceiving a discriminatory work environment and workers’ psychological well-being.

This study makes several contributions. At theoretical level, it improves our understanding about how job resources counteract the negative effects of perceiving a discriminatory work environment. It also showed that the mere presence of job resources is not enough to obtain the best recipe to limit the negative effects of a discriminatory work environment. Resources based interventions might produce the best results only if tailored to workers’ needs.

At a practical level, this study shows that manifesting support is important but not enough for counteracting a discriminatory work environment. People need to perceive a friendly and supporting environment but also they need to perceive that in their organization there is no room for discrimination (Di Marco et al., 2017). In terms of practical implications, job crafting might be a solution to adjust some aspects of the job to workers needs, reducing the levels of demands and/or increasing resources (Tims et al., 2013; van Wingerden and Poell, 2017). For instance, people might craft their interpersonal relationships at work in order to reinforce ties with those people they consider more supportive. Managers should be aware of the positive effects of these interventions, encouraging and facilitating relationship even for those workers who spend time alone due to the nature of their job, as in the case of truck drivers.

Some limitations have to be discussed. Firstly, the cross-sectional design does not allow establishing the causality of mediation (Zapf et al., 1996). Moreover, the low number of sociodemographic variables collected limits the possibility to explore how the moderated mediation model works with different groups of people.

## Conclusion

Experiencing a discriminatory work environment can undermine workers’ psychological well-being. Some job resources, such as job autonomy and social support might reduce its negative effects, but resources based interventions need to be tailored to workers’ needs in order to obtain the best results.

## Ethics Statement

All procedures performed in studies involving human participants were in accordance with the standards of the national law of data treatment followed by the University of Florence (Italy).

## Author Contributions

DDM, AA, GG, GA, and NM equally contributed to all the following issues of the research: conception and design of the work; acquisition, analysis, or interpretation of data for the work; drafting the work and critically revising it; final approval of the version to be published; agreement to be accountable for all aspects of the work in ensuring that questions related to the accuracy or integrity of any part of the work are appropriately investigated and resolved.

## Conflict of Interest Statement

The authors declare that the research was conducted in the absence of any commercial or financial relationships that could be construed as a potential conflict of interest.
